# Risk Factors for Postoperative Fibrinogen Deficiency after Surgical Removal of Intracranial Tumors

**DOI:** 10.1371/journal.pone.0144551

**Published:** 2015-12-11

**Authors:** Naili Wei, Yanfei Jia, Xiu Wang, Yinian Zhang, Guoqiang Yuan, Baotian Zhao, Yao Wang, Kai Zhang, Xinding Zhang, Yawen Pan, Jianguo Zhang

**Affiliations:** 1 Department of Neurosurgery, The Second Hospital of Lanzhou University, Chengguan District, Lanzhou, Gansu, 730030, China; 2 Department of Neurosurgery, Beijing Tiantan Hospital, Capital Medical University, Tiantanxili 6, Dongcheng, Beijing 100050, China; 3 Institute of Neurology, The Second Hospital of Lanzhou University, Chengguan District, Lanzhou, Gansu, 730030, China; The George Washington University, UNITED STATES

## Abstract

Higher levels of fibrinogen, a critical element in hemostasis, are associated with increased postoperative survival rates, especially for patients with massive operative blood loss. Fibrinogen deficiency after surgical management of intracranial tumors may result in postoperative intracranial bleeding and severely worsen patient outcomes. However, no previous studies have systematically identified factors associated with postoperative fibrinogen deficiency. In this study, we retrospectively analyzed data from patients who underwent surgical removal of intracranial tumors in Beijing Tiantan Hospital date from 1/1/2013to12/31/2013. The present study found that patients with postoperative fibrinogen deficiency experienced more operative blood loss and a higher rate of postoperative intracranial hematoma, and they were given more blood transfusions, more plasma transfusions, and were administered larger doses of hemocoagulase compared with patients without postoperative fibrinogen deficiency. Likewise, patients with postoperative fibrinogen deficiency had poorer extended Glasgow Outcome Scale (GOSe), longer hospital stays, and greater hospital expenses than patients without postoperative fibrinogen deficiency. Further, we assessed a comprehensive set of risk factors associated with postoperative fibrinogen deficiency via multiple linear regression. We found that body mass index (BMI), the occurrence of postoperative intracranial hematoma, and administration of hemocoagulasewere positively associated with preoperative-to-postoperative plasma fibrinogen consumption; presenting with a malignant tumor was negatively associated with fibrinogen consumption. Contrary to what might be expected, intraoperative blood loss, the need for blood transfusion, and the need for plasma transfusion were not associated with plasma fibrinogen consumption. Considering our findings together, we concluded that postoperative fibrinogen deficiency is closely associated with postoperative bleeding and poor outcomes and merits careful attention. Practitioners should monitor plasma fibrinogen levels in patients with risk factors for postoperative fibrinogen deficiency. In addition, postoperative fibrinogen deficiency should be remediated as soon as possible to reduce postoperative bleeding, especially when postoperative bleeding is confirmed.

## Introduction

Fibrinogen is synthesized in the liver and plays an essential role in coagulation. As a precursor to clot formation, it strongly affects hemostasis, blood rheology, platelet aggregation and endothelial function. Conversion of fibrinogen to fibrin and formation of an in soluble fibrin clot is the end point of the coagulation pathway[1]. Fibrinogen deficiency results in coagulopathy and is associated with uncontrolled bleeding and compromised survival[2]. For patients with intracranial tumors hemorrhage after surgical removal of tumors might result in adverse events and an increase of morbidity and mortality and severely worsen outcomes[3]. In contrast, high plasma fibrinogen is associated with improved survival rates for patients with massive blood loss[4]. Administration of fibrinogen concentrate improves clotting function and reduces blood loss[5, 6]. Identifying risk factors for fibrinogen deficiency will help practitioners prevent fibrinogen deficiency and will reduce the risk of serious complications, such as postoperative hematoma.

Although fibrinogen is well known for its mechanisms involved in both primary and secondary hemostasis, its functions are far more extensive. So far, plasma fibrinogen level has been reported to be associated with many factors, including patient characteristics such as sex, age and BMI, as well as indicators such as tumor presence, postoperative hematoma, massive bleeding, blood transfusion, plasma transfusion, and administration of hemocoagulase[7–17]. The present study analyzed possible predictors of plasma fibrinogen level to identify risk factors for postoperative fibrinogen deficiency.

## Method

### Study design

**Fig 1:**
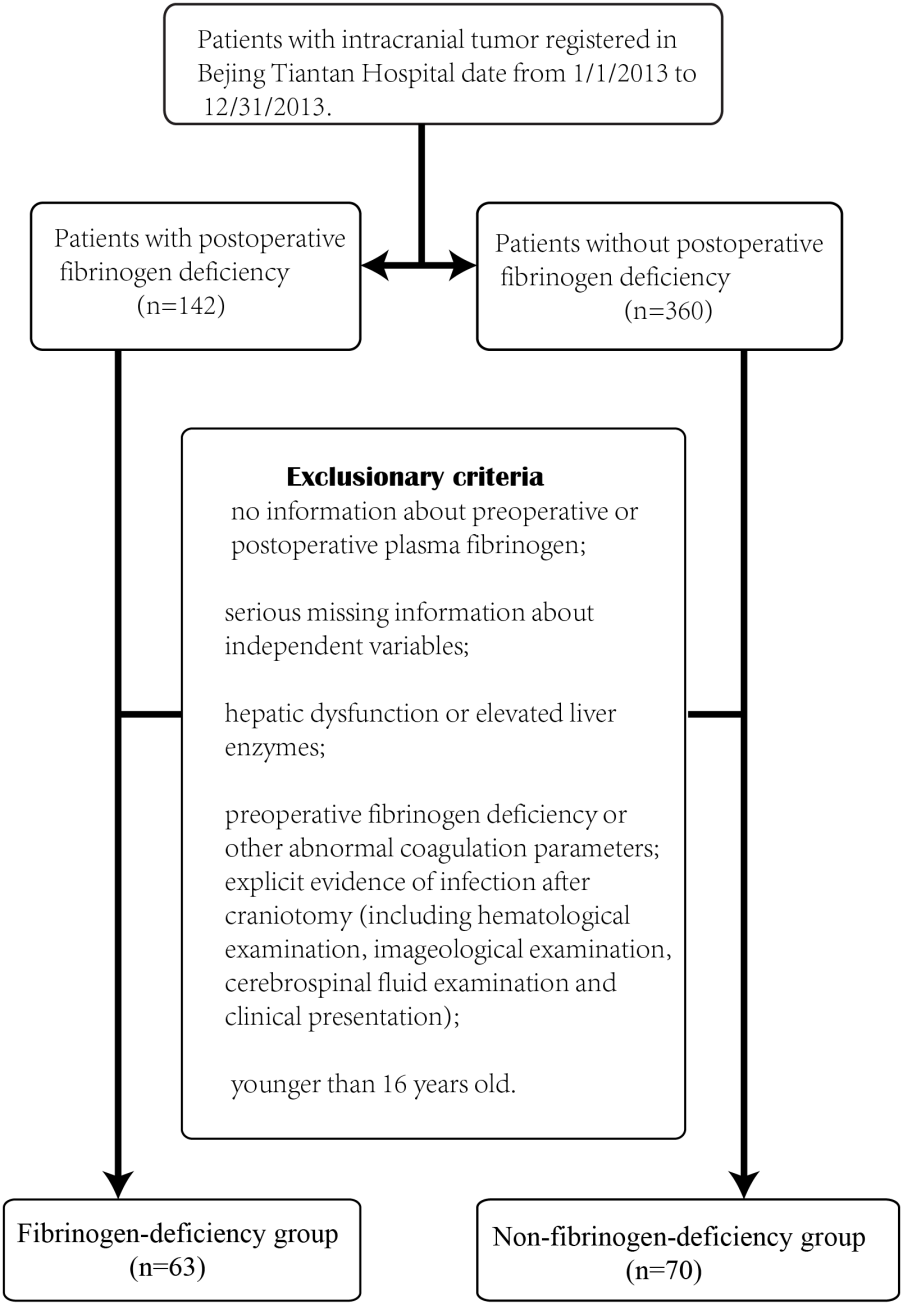
Exclusion criterion.

This study had been approved by the ethics committee of Beijing Tiantan Hospital Affiliated Capital Medical University. Each patient who was enrolled in this study has signed the informed consent. The present study was designed to retrospectively assess potential predictors of plasma fibrinogen level(gender, age, BMI, pathology of tumor, postoperative hematoma, intraoperative blood loss, blood transfusion, plasma transfusion, administration of hemocoagulase) on two groups of patients: a fibrinogen deficiency group and a non-fibrinogen deficiency group. All of the patients enrolled in this study had undergone surgical removal of intracranial tumors in Beijing Tiantan Hospital date from1/1/2013to12/31/2013. The plasma fibrinogen deficiency group was defined as patients having a plasma fibrinogen level<2.0g/l, measured by laboratory assay after operation; the non-deficiency group had fibrinogen levels ≥2.0 g/l. Patient information for the deficiency group(n = 121)was acquired through a database at Beijing Tiantan Hospital through which patients apply for fibrinogen substitute products. An initial sample of potential members of the non-deficiency group (n = 360) was randomly selected from the in-patient database of Beijing Tiantan Hospital. Patients with postoperative fibrinogen deficiency were enrolled in the present study (Fig 1). We reviewed their medical records and enrolled patients who met our exclusion criterion (Fig 1).

The present study first compared all variables between the deficiency and non-deficiency group to identify clinical characteristics of patients with postoperative fibrinogen deficiency. Clinical outcomes of patients between two groups were also compared, including extended Glasgow Outcome Scale (GOSe)[18, 19], length of hospital stay, and hospitalization expenses. Then, we used linear regression models to assess which variables were associated with preoperative-to-postoperative plasma fibrinogen consumption to identify risk factors for postoperative fibrinogen deficiency. In this model, we selected plasma fibrinogen consumption rather than postoperative plasma fibrinogen level as the dependent variable because the former reflects the effect of the variables on plasma fibrinogen level more directly than the latter.

### Statistical analysis

First, we analyzed differences in all variables between the fibrinogen-deficiency group and the non-fibrinogen-deficiency group. Pearson’s chi-squared test or Fisher’s exact test was used to compare differences between the two groups in number of female patients, patients 60 years old or older, patients with malignant tumors and patients experiencing postoperative intracranial hematoma. Independent sample *t*-tests were used to compare the differences between the two groups in pre-operative plasma fibrinogen level, postoperative plasma fibrinogen level, plasma fibrinogen consumption from pre- to post-operation, intraoperative blood loss, amount of plasma transfusion, amount of blood transfusion, and dose of hemocoagulase administered. Clinical outcomes between the two groups were also compared via independent sample t-tests, including length of hospital stay, and hospitalization expenses. The GOSe of the two groups was compared by Mean-Whitney U test.

Second, using multiple linear regression, we assessed variables (gender, age, BMI, malignant tumor, postoperative intracranial hematoma, intraoperative blood loss, blood transfusion, plasma transfusion, dose of hemocoagulase administered) that might be associated with plasma fibrinogen consumption (Table 1).

**Table 1:** Variables.

**Variable**	**Dump variable**
**Dependent variable (Y)**	
Plasma fibrinogen consumption(from preoperative to postoperative level)	
**Independent variable (X)**	
BMI	
Gender	0: male; 1: female.
Old age	0: younger than 60 years old; 1: 60 or older than 60 years old.
Pathologic type of tumor	0: benign; 1: malignant.
Postoperative intracranial hematoma after operation	0: no; 1: yes.
Blood loss in operation	
Plasma transfusion	
Blood transfusion	
Hemocoagulase administration	

BMI: Body mass index.

## Results

The present study ultimately included 63 patients in the fibrinogen-deficiency group and 70 patients in the non-fibrinogen-deficiency group. As Table 2 shows, compared with patients without fibrinogen deficiency, patients with postoperative fibrinogen deficiency had a higher rate of postoperative intracranial hematoma (χ^2^_0.05,1_ = 14.708, p<0.001). They had more intraoperative blood loss (t = 23.695, p = 0.005) and more plasma fibrinogen consumption from preoperative to postoperative level (t = 9.668, p<0.001). Accordingly, they were given more blood transfusion (t = 24.997, p = 0.005), plasma transfusion(t = 14.275, p = 0.024) and were administered larger doses of hemocoagulase(t = 19.362, p = 0.007) (Table 2). Patients with postoperative fibrinogen deficiency had poorer outcomes than those without postoperative fibrinogen deficiency. As Table 3 shows, patients with postoperative deficiency had poorer GOSe, longer hospital stays, and higher hospitalization expenses than patients in the non-fibrinogen deficiency group.

**Table 2:** Differences in clinical characteristics between fibrinogen deficiency group and non-fibrinogen deficiency group.

	Fibrinogen deficiency group	Non-fibrinogen deficiency group	F/χ^2^_0.05,1_	Pvalue
**patients**	n = 63	n = 70		
Preoperative plasma fibrinogen level	2.86±0.081	3.051±0.079	0.039	0.088 ^a^
Postoperative plasma fibrinogen level	1.343±0.063	3.256±0.113	20.439	<0.001 ^a^
Plasma fibrinogen consumption	1.513±0.117	-0.205±0.134	0.360	<0.001 ^a^
BMI	23.792±0.458	23.077±0.375	0.721	0.229 ^a^
Female	29	31	0.760	0.862 ^b^
Old age	12	17	0.534	0.532^b^
Malignant tumor	14	20	0.702	0.432^b^
Postoperative intracranial hematoma	18	3	14.708	<0.001^b^
Intraoperative blood loss	1385.714±333.265	479.571±46.800	23.695	0.005 ^a^
Plasma transfusion	464.762±157.531	115.463±31.061	14.275	0.024 ^a^
Blood transfusion (ml)	291.746±85.096	47.286.±16.523	24.997	0.004 ^a^
Dose of hemocoagulase administration (U)	7.667±1.166	4.186±0.572	19.362	0.007 ^a^

^a^: comparison via independent samples *t*-test;

^b^: comparison via Pearson’s chi-squared test or Fisher’s exact test. The results were presented as the mean±SEM.

**Table 3:** Outcomes between fibrinogen deficiency group and non-fibrinogen deficiency group.

**Outcome**	**Fibrinogen deficiency group**	**Non-fibrinogen deficiency group**	**F/Z**	**P value**
**patients**	N = 63	N = 70		
**GOSe(1–8 point)**			-1.968	0.049 a
Good recovery; upper(8)	41	55		
Good recovery; lower(7)	10	10		
Moderately disabled; upper(6)	4	3		
Moderately disabled; lower(5)	2	1		
Severely disabled; upper(4)	1	0		
Severely disabled; lower(3)	1	1		
Persistent vegetative state(2)	2	0		
Dead(1)	2	0		
**hospital stay(d)**	24.746±2.041	17.171±0.879	9.270	0.001 b
**hospital expense(¥)**	124649.672±21391.862	74498.486±13978.170	6.253	0.048 b

^a^: comparisonvia Mean-Whitney U test;

^b^: comparison via independentsamples *t*-test.GOSe: extended Glasgow Outcome Scale. The results were presented as the mean±SEM.

When the variables were analyzed with multiple linear regression, intraoperative blood loss, blood transfusion and plasma transfusion were not associated with fibrinogen consumption. Instead, BMI(β = 0.075, p = 0.018), postoperative intracranial hematoma(β = 0.664, p = 0.0.03), and dose of hemocoagulase administered(β = 0.064, p<0.001)were positively associated with plasma fibrinogen consumption (Table 4). Interestingly, having a malignant tumor (β = -0.565, p = 0.023) was negatively associated with plasma fibrinogen consumption (Table 4).

**Table 4:** Risk factors associated with plasma fibrinogen consumption from preoperative to postoperative level.

Variable	β	P value
Constant	-1.567	0.044
BMI	0.075	0.019
Female	0.173	0.418
Old age	-0.213	0.408
Malignant tumor	-0.565	0.023
Intracranial hematoma after operation	0.664	0.030
Blood loss in operation(ml)	2.72E-6	0.988
Plasma transfusion(ml)	0.000	0.323
Blood transfusion (ml)	0.001	0.098
Hemocoagulase administration (U)	0.064	<0.001

## Discussion

Fibrinogen plays a critical role in the process of hemostasis: it facilitates platelet aggregation to form a spider’s web in the initial platelet plug, and cleavage by thrombin forms clots[5]. Increasing fibrinogen concentration increases clot stability[5]. However, decreasing plasma fibrinogen level can increase the risk of bleeding. When fibrinogen levels decrease below 2.0 g/l, the risk for bleeding increases10-fold[12]. Fibrinogen deficiency results in coagulopathy and is associated with uncontrolled bleeding[2]. The present study indicated that patients with postoperative fibrinogen deficiency had worse outcomes than patients with higher plasma fibrinogen levels. The present study also revealed that plasma fibrinogen consumption was highly associated with postoperative intracranial hematoma, consistent with previous work[12]. Postoperative intracranial bleeding was associated with worsened outcomes, increased mortality and disability, prolonged length of stay and increased hospital expenses. Thus, postoperative fibrinogen deficiency had a close relationship with postoperative intracranial bleeding and patient outcomes, and it deserves neurosurgeons’ attention. Practitioners should regularly monitor patients’ plasma fibrinogen level in the 48 hours after surgery, the peak period of postoperative bleeding. If fibrinogen deficiency is detected, it should be addressed immediately. Although a threshold level of 1.0 g/L has been recommended for fibrinogen supplementation in trauma patients, a minimum threshold of 2.0 g/L has been identified in vitro for the optimal rate of clot formation[6]. In addition, fibrinogen supplementation increases clot strength regardless of plasma fibrinogen level[6] and reduces postoperative intracranial bleeding. Therefore, in our perspective, a threshold of 2.0 g/l is recommended for postoperative fibrinogen supplementation.

Postoperative intracranial hematoma could consume additional plasma fibrinogen and accelerate decreases in plasma fibrinogen: more fibrinogen would be consumed to form fibrinogen clot. However, one weakness of our study is that it cannot answer the question of whether hematoma-induced clot formation resulted in fibrinogen deficiency or fibrinogen deficiency resulted in hematoma. Resolving this question requires identifying whether clot formation or fibrinogen deficiency occurs first. Fibrinogen is not routinely monitored during treatment. It is difficult to guarantee that head CT scan and postoperative fibrinogen level were examined at the same point across patients. Further exploration of this issue may require a prospective study.

Hemocoagulase (batroxobin) is administered in large doses in clinical treatment to promote blood clotting and prevent postoperative bleeding. Its effect has been associated with a reduction of fibrinogen levels in plasma and an enhancement of anticoagulation and fibrinolysis[17],[20, 21]. Fibrinogen facilitates the aggregation of platelets and is cleaved by thrombin to produce fibrin monomers, which polymerize to form clots[22]. Hemocoagulase promotes fibrinogen clot formation and lowers circulating fibrinogen level[20]. Although administration of hemocoagulase is safe and serious adverse events are infrequent[17], some papers have reported that long-term administration of hemocoagulase leads to fibrinogen deficiency and postoperative bleeding[23], which is consistent with our findings. Long-term administration of hemocoagulase increases plasma fibrinogen consumption and thus results in postoperative fibrinogen deficiency. Our findings revealed that patients with massive intraoperative blood loss received larger doses of hemocoagulase to reduce intra- or postoperative bleeding. Some patients enrolled in our study received hemocoagulase on more than 5 days. However, surgeons may be unaware that long-term or large-dose administration of hemocoagulase could result in fibrinogen deficiency and increase the risk of postoperative bleeding. The minimum plasma fibrinogen level required for the optimal rate of clot formation is 2.0 g/l.[24] Hemocoagulase use may be unwise for patients with fibrinogen deficiency. Our study indicated that administration of hemocoagulase was a risk factor for postoperative fibrinogen deficiency. Whereas hemocoagulase is typically administered with the assumption that plasma fibrinogen levels are normal, doctors must recognize that patients with massive blood loss may have reduced plasma fibrinogen levels, especially when postoperative bleeding was confirmed.

Existing work demonstrates that plasma fibrinogen level is positively associated with BMI[9, 25–27]. The present study extends our understanding by showing that obesity is positively associated with decreases in plasma fibrinogen after surgery. These findings suggest that patients with higher BMI needed to consume more plasma fibrinogen to clot. Disturbances in hemostatic and fibrinolytic systems have previously been reported in obese patients[8]. We postulate that the clotting function of plasma fibrinogen in obese patients declined. When massive blood loss occurred intraoperatively, more plasma fibrinogen might be consumed to form clots. Therefore, obese patients are at risk for postoperative fibrinogen deficiency.

Fibrinogen has also been reported to be associated with tumor metastasis, although the linking mechanism is unclear[13, 28]. Tumor cells could promote fibrinogen synthesis and fibrinogen could deposit on tumor cells, serving as a scaffold to support binding of growth factors to promote tumor cell growth and metastasis[15, 29]. Our study found that removal of a malignant tumor was negatively associated with plasma fibrinogen consumption—that is, resection of malignant tumor increased plasma fibrinogen level. We postulate that large amounts of fibrinogen from tumor cells were released into blood circulation and thus increased plasma fibrinogen level during brain tumor resection. This is consistent with previous evidence that neurosurgical operations for intracerebral primary tumors resulted in hypercoagulability syndrome[30]. Therefore, we suggest that benign tumors indicate greater risk of postoperative fibrinogen deficiency than do malignant tumors.

Previous work has demonstrated that plasma concentrations of clotting factors including fibrinogen significantly decrease after surgery because of blood loss and colloid or crystalloid fluid dilution[31]. Blood transfusion and plasma transfusion have also been reported to enhance plasma fibrinogen level[32, 33]. Although our findings showed that patients with postoperative fibrinogen deficiency had more intraoperative blood loss, multiple linear regression results revealed that plasma fibrinogen was not associated with blood loss. Neither blood transfusion nor plasma transfusion was associated with fibrinogen consumption. We attribute these surprising results to the fact that patients with massive blood loss were spontaneously given blood transfusion, plasma transfusion and homeostasis procedures. The present study cannot distinguish the effect of blood loss on plasma fibrinogen consumption from the effects of blood transfusion and plasma transfusion. This point highlights a disadvantage of the present study’s retrospective nature.

In conclusion, postoperative fibrinogen deficiency was closely related to postoperative bleeding and poor outcomes and deserves practitioners’ attention. Obesity, postoperative intracranial hematoma, and higher doses of hemocoagulase were risk factors for postoperative fibrinogen deficiency. Having a benign tumor increased the risk for postoperative fibrinogen deficiency relative to the risk associated with having a malignant tumor. We recommend monitoring plasma fibrinogen levels for patients with risk factors for postoperative fibrinogen deficiency. In addition, postoperative fibrinogen deficiency should be remedied as soon as possible to reduce postoperative bleeding, especially when postoperative bleeding is confirmed.

## Supporting Information

S1 Table Fibrinogen deficiency group data. (XLSX)

S2 Table Non-fibrinogen-deficiency group data. (XLSX)

S3 Table Linear regression model data. (XLSX)
